# Differences in physical activity participation among young adults in Aotearoa New Zealand

**DOI:** 10.1186/s12889-023-15063-6

**Published:** 2023-01-23

**Authors:** Oliver W. A. Wilson, Melody Smith, Scott Duncan, Erica Hinckson, Anja Mizdrak, Justin Richards

**Affiliations:** 1 Te Hau Kori, Faculty of Health, Victoria University of Wellington, PO Box 600, Wellington, 6140 New Zealand; 2 School of Nursing, Faculty of Medical and Health Sciences, the University of Auckland, Private Bag 92019, Auckland, 1142 New Zealand; 3 School of Sport and Recreation, Faculty of Health and Environmental Sciences, Auckland University of Technology, Private Bag 92006, Auckland, 1142 New Zealand; 4 Department of Public Health, University of Otago, PO Box 7343, Wellington, 6242 New Zealand; 5 Sport New Zealand Ihi Aotearoa, Wellington, New Zealand

**Keywords:** Exercise, Health, Gender, Ethnicity, Disability, Socio-economic status

## Abstract

**Background:**

The purpose of this study was to examine socio-demographic differences in physical activity (aerobic and muscle-strengthening) among young adults (18–24 years).

**Methods:**

Data collected between 2017–2019 as a part of Sport New Zealand’s Active NZ survey were examined using logistic regression analyses to determine the odds of participants meeting aerobic, muscle-strengthening and combined physical activity recommendations. Gender, ethnicity, employment/student status, disability status, and socio-economic deprivation were included as explanatory variables in analyses.

**Results:**

The proportion of young adults meeting recommendations varied according to physical activity type (aerobic:63.2%; strength:40.1%; combined:37.2%). Young adults not employed/studying had lower odds of meeting recommendations than those full-time employed (OR = 0.43 [0.34–0.54]). Physical activity levels differ according to gender and this intersects with ethnicity, employment/student status, and social deprivation. For example, the odds of Pasifika young adults meeting combined physical activity recommendations compared to Europeans were not different (OR = 0.95 [0.76–1.19]), but when stratified by gender the odds were significantly higher for men (OR = 1.55 [1.11–2.16]) and significantly lower for women (OR = 0.64 [0.47–0.89]. Similarly, young adults in high deprivation areas had lower odds of meeting combined physical activity recommendations than those in low deprivation areas (OR = 0.81 [0.68–0.95]), but this was mainly due to the difference among women (OR = 0.68 [0.54–0.85]) as there was no difference among men (OR = 0.97 [0.76–1.25]).

**Conclusions:**

Intersections between socio-demographic characteristics should be considered when promoting physical activity among young adults in Aotearoa New Zealand, particularly young adults not employed/studying, and young women who live in deprived areas or identify as Asian or Pasifika. Tailored approaches according to activity type for each of these groups are required.

## Background

While physical activity among children and youth in Aotearoa New Zealand (NZ) is collated and reported on regularly [1], relatively little is known about the physical activity behaviours of young adults (i.e. 18–24 year-olds). It is widely recognised that several lifelong health behaviours are embedded during young adulthood [2]. There is also international evidence suggesting that there is typically a decline to relatively low levels of physical activity during this period [3, 4], which continues a decline that is begins in later adolescence among young people in NZ when there is a shift from most young people meeting physical activity recommendations to less than half [5]. This is important because physical activity, both aerobic and muscle-strengthening, is associated with various physical and mental health benefits [6–8]. Similar to several other health behaviours, participation in physical activity across the lifespan varies according to a variety of socio-demographic characteristics (e.g. gender, age, ethnicity, sexual orientation, socio-economic status, [dis]ability) [9, 10]. Inequities in physical activity participation tend to follow a social gradient, with less advantaged individuals typically less physically active and therefore more likely to experience adverse associated health outcomes [11]. Such inequities based on gender, ethnicity, deprivation, and disability status are evident among adults in NZ [12]. Thus, identifying factors that contribute to physical activity participation differences is important for developing and implementing tailored interventions to address them [13, 14].

Physical activity participation differences among adults are typically reported in the grey literature as descriptive statistics based on data for aerobic activity only and stratified according to sex, ethnicity, deprivation, or age [15–17]. There are numerous shortcomings to this approach. Firstly, the focus on aerobic activity does not account for differences in participation in different types of activity, which may vary in prevalence and importance according to socio-demographics (e.g. muscle strengthening in older adults). Secondly, there are differences in how different categories within these socio-demographic characteristics are defined and reported (e.g. gender vs. sex). Thirdly, this approach does not parse differences in a way that accounts for intersections amongst them (e.g. deprivation and ethnicity). Finally, several characteristics that may also contribute to inequitable outcomes are not typically included (e.g. [dis]ability and employment). Consequently, there is limited international evidence regarding differences for physical activity participation in both aerobic and muscle-strengthening activity based on multiple socio-demographic characteristics, particularly among young adults.

The aim of this study is to identify differences in physical activity participation, both aerobic and muscle-strengthening, among young adults in NZ, taking into consideration a broad range of known socio-demographic correlates for participation [18]. This will provide better insight into the underlying factors driving inequitable participation in physical activity [19, 20]. In doing so, our objective is to demonstrate the value of more nuanced analyses of physical activity surveillance data beyond the descriptive statistic stratification commonly used for describing population-level inequities globally. More locally, we also endeavour to provide clear guidance on future intervention priorities to promote physical activity in the young adult population of NZ.

## Methods

Data from 8,598 individuals were collected as a part of the Active NZ survey across 12 quarterly waves between January 2017 and December 2019. A multi-stage probability sampling procedure was applied to recruit participants selected at the household level from the NZ Electoral Role. Full details of the survey methods are articulated in the annual Active NZ Technical reports [21–23], but a summary of key components is outlined below.

### Participants

Adults (aged ≥ 18 years) were recruited to participate in the survey using the NZ electoral roll as a sampling frame. Participants were contacted via mail and invited to complete the survey online or using a paper questionnaire. Response rates over the three years averaged 31.1%.

### Data collection

The self-report survey comprised items about socio-demographic characteristics and physical activity behaviours. Data were collected continuously throughout the year to account for seasonal variation in physical activity participation.

### Measures

#### Age

Participants specified their age group using categories, starting at 18–19 years, followed by 20–24 years, with subsequent age categories increasing in five-year increments.

#### Gender

Participants identified their gender (male, female, or gender diverse). Due to small number of participants identifying as gender diverse (*n* = 46), we excluded these participants from our analyses.

#### Ethnicity

Participants identified their ethnic group(s) and there was no limit on the number of ethnicities they could choose. For the purposes of these analyses, participants who identified multiple ethnicities were categorised to only one of these in the following order: Māori, Pasifika, Asian, Middle Eastern / Latin American / African (MELAA), European, Other. Due to small number of participants identifying as an ‘other’ ethnicity (*n* = 20), we excluded these participants from our analyses.

#### Employment / student status

Participants were asked to respond (yes / no) to items concerning their situation with respect to work and educational pursuits. Participants were categorised into those who worked full-time, part-time, or who were unemployed. Participants were also categorised into full-time students (post-secondary and secondary) or non-students. Those reporting they were part-time students were considered non-students for the purposes of this analyses. Based on combining the above categories, participants were categorised into one of five groups: 1) Full-time worker/non-students; 2) Part-time worker/non-student; 3) not in employment, education, or training (NEET) [24]; 4) full-time student/non-worker; 5) full-time student/worker.

#### Disability status

Participants who did not report using a wheelchair, using a walking aid, using prosthetics, or dealing with an ongoing physical illness were classified as non-disabled.

#### Deprivation status

Deprivation was determined using the 2018 NZ Index of Deprivation, which combines census data relating to income, home ownership, employment, qualifications, family structure, housing, access to transport and communications to designate small geographic areas (60–110 people) with a decile number ranging from 1 (least deprived) to 10 (most deprived) [25]. Participants were classified as residing in low (deciles 1–3), medium (deciles 4–7) and high (deciles 8–10) deprivation areas.

#### Physical activity

Participants self-reported the total duration of their participation in physical activities in the past seven days for sport, exercise, or recreation. This was then dichotomised into those who did / not meet aerobic physical activity recommendations (≥ 150 min/week) [26].

Participants self-reported whether they participated in muscle-strengthening activities on at least two days in the past 7 days (yes / no). Participants were also dichotomised into those who did / not meet both aerobic and muscle-strengthening activity recommendations [26].

### Data analyses

Analyses were conducted on individuals aged between 18 and 24 years of age at the time of completing the survey. All analyses were completed using data weighted according to NZ census population distribution for age, gender, ethnicity, income, household size and geographical region. Data for muscle-strengthening recommendations were missing for part of the sample, and as such the sample used to examine differences in muscle-strengthening and combined physical activity recommendations was smaller than that used to examine differences in aerobic physical activity. Descriptive statistics were used to compute the percentage of young adults meeting physical activity (aerobic, muscle-strengthening, and combined physical activity) recommendations in line with those of the World Health Organization [26]. Logistic regression analyses were used to compute odds ratios of young adults meeting aerobic activity recommendations, muscle-strengthening recommendations, and both aerobic and muscle-strengthening recommendations. Regression analyses are reported for both unadjusted (i.e. single demographic characteristic) and adjusted (i.e. all demographic characteristics) models. Further regression analyses were separated by gender to allow for the intersection between gender and other socio-demographic characteristics. Inconsistencies in the results for the unadjusted vs. adjusted models were identified and the odds ratios (OR) from the fully adjusted models were used to report differences and formulate recommendations. All analyses used SPSS 28.0 (IBM, Armonk, NY), with significance levels set at *p* < 0.05.

## Results

### Participant characteristics

Participant characteristics are displayed in Table 1. Participants who did not specify gender and/or ethnicity, or for whom deprivation status could not be determined were excluded from analyses (*n* = 1330). Sensitivity analysis indicated that these excluded participants were not significantly different to the included sample for any of the physical activity outcomes of interest. Analyses were conducted on the remaining 7,248 participants unless stated otherwise. Less than 40% of young adults met both aerobic and muscle-strengthening recommendations, but there was substantial variation across different socio-demographic groups. The proportion of the population meeting the muscle-strengthening recommendations was substantially lower than that meeting aerobic populations. Although this was the case across all socio-demographics included in the subsequent models, there was variation in the magnitude of this difference across groups.

**Table 1:** Participant characteristics

	Sample (*n* = 7248)		Meeting aerobic recommendations (*n* = 7237)		Meeting muscle-strengthening recommendations (*n* = 4190)		Meeting physical activity recommendations (*n* = 4190)	
	n	%	n	%	n	%	n	%
**Gender**								
Men	2906	40.1	1897	65.4	710	41.7	667	39.2
Women	4342	59.9	2676	61.7	970	39.0	892	35.9
Gender diverse^a^	46		23	50.2	10	37.1	9	34.3
**Ethnicity**								
European	5131	70.8	3362	65.6	1200	41.0	1132	38.7
Māori	903	12.5	565	62.6	197	40.0	183	37.1
Pasifika	330	4.6	158	48.2	71	36.8	61	31.6
Asian	808	11.1	450	55.7	191	36.0	168	31.7
MELAA	76	1.0	38	50.0	21	44.7	15	31.9
Other^a^	20		9	45.0	7	53.8	5	38.5
**Employment/student status**								
Work full-time (non-student)	2615	36.1	1703	65.2	667	44.2	632	41.9
Work part-time (non-student)	899	12.4	525	58.6	186	35.5	171	32.6
NEET	803	11.1	399	49.8	121	27.0	105	23.4
Full-time student (non-worker)	1681	23.2	1106	65.9	398	41.7	364	38.1
Full-time student (worker)	1250	17.2	840	67.3	308	40.8	287	38.0
**Ability status**								
Non-disabled	6666	92.0	4250	63.9	1569	40.8	1458	37.9
Disabled	582	8.0	323	55.6	111	32.6	101	29.6
**Deprivation status**								
Low	2793	38.5	1894	67.9	690	42.1	649	39.6
Medium	2839	39.2	1791	63.1	657	40.0	610	37.1
High	1616	22.3	888	55.2	333	36.6	300	33.0
**OVERALL**			4582	63.2	1680	40.1	1559	37.2

^a^Gender diverse, and ‘other’ ethnicity statistics were calculated using raw weighted data and were excluded from subsequent analyses due to limited sample size; Middle Eastern / Latin American / African = MELAA; Not in employment, education, or training = NEET

### Aerobic physical activity

Differences in aerobic activity based on socio-demographic characteristics are displayed in Table 2. Adjusted and unadjusted models were consistent, except men who were NEET and women who were full-time students/non-workers. Specifically, in the unadjusted model men who were NEET had significantly lower odds of meeting aerobic recommendations that full time workers/non students, but this difference was not significant in the adjusted model. Similarly, in the unadjusted model women who were full time students/non workers had significantly lower odds of meeting aerobic recommendations than the referent group to, but this difference was not significant adjusted model. Overall, women had 18% lower odds of meeting aerobic recommendations compared to men. Pasifika and Asian people both had 33% lower odds of meeting aerobic recommendations compared to Europeans. When stratified by gender, lower odds for meeting aerobic recommendations were observed for Asian men (25% lower), Asian women (41% lower) and Pasifika women (45% lower). Compared to those in full-time employment who were non-students, people who were NEET had 38% lower odds overall and women who were NEET had 50% lower odds of meeting the aerobic recommendations. Part-time employment was also associated with lower odds of meeting aerobic recommendations overall (14% lower) and for women (28% lower). In contrast, men who were full-time students had 30% higher odds of meeting aerobic recommendations if they were not also employed and 47% higher odds if they did have concurrent employment compared to men who were in full-time employment and non-students. No significant differences overall, or among either gender, were evident based on disability status. Overall, the odds of meeting aerobic recommendations were lower among those residing in high deprivation communities when compared to low deprivation communities (medium deprivation: 16% lower, high deprivation: 32% lower). This was consistent with findings among women (medium deprivation: 24% lower, high deprivation: 34% lower), but only men in high deprivation communities had significantly lower odds of meeting aerobic recommendations (30% lower).

**Table 2:** Differences in meeting aerobic physical activity recommendations (*n* = 7237)

	Overall		Men		Women	
	Unadjusted	Adjusted	Unadjusted	Adjusted	Unadjusted	Adjusted
	OR (95%CI)					
Men (Referent)	1.00	1.00				
Women	**0.81 (0.74–0.88)**	**0.82 (0.75–0.90)**				
European (Referent)	1.00	1.00	1.00	1.00	1.00	1.00
Māori	0.90 (0.78–1.04)	1.05 (0.91–1.22)	0.86 (0.68–1.08)	0.95 (0.75–1.21)	0.94 (0.78–1.13)	1.12 (0.93–1.35)
Pasifika	**0.54 (0.46–0.63)**	**0.67 (0.57–0.80)**	0.81 (0.63–1.05)	0.97 (0.74–1.26)	**0.42 (0.35–0.52)**	**0.55 (0.44–0.68)**
Asian	**0.68 (0.60–0.77)**	**0.67 (0.59–0.77)**	**0.78 (0.65–0.94)**	**0.75 (0.62–0.91)**	**0.59 (0.49–0.70)**	**0.59 (0.49–0.71)**
MELAA	0.65 (0.42–1.00)	0.68 (0.43–1.07)	0.52 (0.24–1.11)	0.52 (0.24–1.12)	0.74 (0.43–1.26)	0.76 (0.44–1.31)
Full-time worker/non-student (Referent)	1.00	1.00	1.00	1.00	1.00	1.00
Part-time worker/non-student	**0.82 (0.70–0.95)**	**0.86 (0.73–1.00)**	0.98 (0.78–1.26)	1.01 (0.79–1.31)	**0.74 (0.61–0.90)**	**0.72 (0.59–0.88)**
NEET	**0.54 (0.47–0.63)**	**0.62 (0.53–0.72)**	**0.75 (0.60–0.95)**	0.82 (0.65–1.04)	**0.43 (0.35–0.52)**	**0.50 (0.40–0.61)**
Full-time student/non-worker	0.99 (0.88–1.12)	1.06 (0.93–1.20)	**1.26 (1.07–1.51)**	**1.30 (1.08–1.57)**	**0.81 (0.68–0.95)**	0.89 (0.75–1.06)
Full-time student/worker	1.10 (0.96–1.27)	1.15 (1.00–1.33)	**1.43 (1.14–1.79)**	**1.47 (1.17–1.85)**	0.95 (0.79–1.13)	0.97 (0.81–1.16)
Non-disabled (Referent)	1.00	1.00	1.00	1.00	1.00	1.00
Disabled	0.84 (0.71–1.00)	0.87 (0.73–1.03)	0.77 (0.58–1.01)	0.77 (0.58–1.03)	0.91 (0.74–1.13)	0.91 (0.74–1.13)
Low deprivation (Referent)	1.00	1.00	1.00	1.00	1.00	1.00
Medium deprivation	**0.80 (0.72–0.89)**	**0.84 (0.76–0.94)**	0.90 (0.77–1.05)	0.95 (0.82–1.13)	**0.34 (0.64–0.85)**	**0.76 (0.66–0.88)**
High deprivation	**0.58 (0.51–0.65)**	**0.68 (0.60–0.77)**	**0.64 (0.54–0.77)**	**0.70 (0.58–0.85)**	**0.54 (0.47–0.63)**	**0.66 (0.56–0.78)**

Middle Eastern / Latin American / African = MELAA; Not in employment, education, or training = NEET; Unadjusted analyses included socio-demographic characteristics in separate models, whereas the adjusted analyses included socio-demographic characteristics in a single model

^*^Bold text indicate significance levels *p* < 0.05

### Muscle-strengthening activity

Differences in muscle-strengthening activity based on socio-demographic characteristics are displayed in Table 3. Adjusted and unadjusted models were consistent, except for the following groups who were less likely meet muscle-strengthening recommendations in the unadjusted, but not adjusted models: women overall, women of Pasifika ethnicity, women who were full-time students/non-workers, people living in medium and high deprivation areas. Overall, Asian people had 24% lower odds of meeting muscle-strengthening recommendations compared to Europeans and for Asian women the odds were 53% lower. In contrast, Pasifika men had 44% higher odds of meeting muscle-strengthening recommendations. People who were NEET had lower odds of meeting muscle-strengthening recommendations than those in full-time employment (overall: 52% lower, men: 30% lower, women: 65% lower). Part-time employment was also associated with lower odds of meeting muscle-strengthening recommendations overall (31% lower) and for women (44% lower). No significant differences overall, or among either gender, were evident based on disability status. Among women, the odds of meeting muscle-strengthening recommendations were 25% lower for those living in medium deprivation and 24% lower for those living in high deprivation when compared to those living in low deprivation communities.

**Table 3:** Differences in meeting muscle-strengthening physical activity recommendations (*n* = 4190)

	Overall		Men		Women	
	Unadjusted	Adjusted	Unadjusted	Adjusted	Unadjusted	Adjusted
	OR (95%CI)					
Men (Referent)	1.00	1.00				
Women	**0.86 (0.77–0.97)**	0.89 (0.79–1.00)				
European (Referent)	1.00	1.00	1.00	1.00	1.00	1.00
Māori	0.93 (0.78–1.12)	1.03 (0.86–1.25)	1.17 (0.87–1.56)	1.18 (0.87–1.59)	0.81 (0.64–1.02)	0.95 (0.75–1.21)
Pasifika	0.89 (0.72–1.09)	1.01 (0.81–1.26)	**1.44 (1.05–1.96)**	**1.44 (1.04–2.01**	**0.62 (0.47–0.82)**	0.78 (0.58–1.01)
Asian	**0.76 (0.65–0.90)**	**0.76 (0.64–0.90)**	1.15 (0.92–1.45)	1.12 (0.89–1.42)	**0.47 (0.36–0.61)**	**0.47 (0.36–0.61)**
MELAA	1.18 (0.67–2.08)	1.28 (0.72–2.26)	2.32 (0.86–6.28)	2.36 (0.87–6.44)	0.83 (0.41–1.68)	0.92 (0.45–1.89)
Full-time worker/non-student (Referent)	1.00	1.00	1.00	1.00	1.00	1.00
Part-time worker/non-student	**0.68 (0.56–0.83)**	**0.69 (0.57–0.85)**	0.89 (0.65–1.22)	0.96 (0.63–1.17)	**0.56 (0.44–0.73)**	**0.56 (0.43–0.73)**
NEET	**0.46 (0.37–0.57)**	**0.48 (0.39–0.60)**	**0.73 (0.53–1.00)**	**0.70 (0.50–0.97)**	**0.32 (0.24–0.43)**	**0.35 (0.26–0.47)**
Full-time student/non-worker	0.95 (0.81–1.10)	0.98 (0.84–1.15)	1.24 (0.99–1.54)	1.24 (0.99–1.55)	**0.73 (0.59–0.91)**	0.80 (0.64–1.00)
Full-time student/worker	0.91 (0.77–1.07)	0.94 (0.79–1.11)	1.04 (0.80–1.35)	1.04 (0.80–1.36)	0.80 (0.64–1.00)	0.84 (0.67–1.05)
Non-disabled (Referent)	1.00	1.00	1.00	1.00	1.00	1.00
Disabled	0.82 (0.66–1.03)	0.89 (0.71–1.12)	0.86 (0.60–1.23)	0.93 (0.65–1.35)	0.82 (0.62–1.09)	0.83 (0.62–1.12)
Low deprivation (Referent)	1.00	1.00	1.00	1.00	1.00	1.00
Medium deprivation	**0.87 (0.76–0.99)**	0.91 (0.79–1.04)	1.08 (0.89–1.31)	1.09 (0.90–1.33)	**0.73 (0.61–0.88)**	**0.75 (0.62–0.90)**
High deprivation	**0.82 (0.70–0.95)**	0.90 (0.76–1.06)	1.16 (0.92–1.46)	1.11 (0.86–1.42)	**0.63 (0.52–0.78)**	**0.76 (0.61–0.94)**

Middle Eastern / Latin American / African = MELAA; Not in employment, education, or training = NEET; Unadjusted analyses included socio-demographic characteristics in separate models, whereas the adjusted analyses included socio-demographic characteristics in a single model

^*^Bold text indicate significance levels *p* < 0.05

### Combined aerobic and muscle-strengthening physical activity

Differences in physical activity (aerobic and muscle-strengthening activity combined) based on socio-demographic characteristics are displayed in Table 4. Adjusted and unadjusted models were consistent, except women, people of Pasifika ethnicity, and those living in medium deprivation communities were less likely to meet combined recommendations in the overall unadjusted, but not adjusted models. Overall, Asian people had 27% lower odds of meeting combined recommendations compared to Europeans. When stratified by gender, lower odds for meeting combined recommendations were observed for Asian women (55% lower) and Pasifika women (36% lower). In contrast, Pasifika men had 55% higher odds of meeting combined recommendations. People who were NEET had lower odds of meeting combined recommendations compared to those in full-time employment (overall: 57% lower, men: 31% lower, women: 71% lower). Part-time employment was also associated with lower odds of meeting combined recommendations overall (31% lower) and for women (48% lower). Women who were full-time students also had 21% lower odds of meeting combined recommendations compared to those in full-time employment. No significant differences overall, or among either gender, were evident based on disability status. Overall, the odds of meeting combined recommendations were 19% lower for those living in high deprivation compared to those living in low deprivation communities. Among women, the odds of meeting combined recommendations was lower among those residing in higher deprivation communities (medium deprivation: 29% lower, high deprivation: 32% lower).

**Table 4:** Differences in meeting both aerobic and muscle-strengthening physical activity recommendations (*n* = 4190)

	Overall		Men		Women	
	Unadjusted	Adjusted	Unadjusted	Adjusted	Unadjusted	Adjusted
	OR (95%CI)					
Men (Referent)	1.00	1.00				
Women	**0.86 (0.76–0.96)**	0.89 (0.79–1.01)				
European (Referent)	1.00	1.00	1.00	1.00	1.00	1.00
Māori	0.91 (0.76–1.10)	1.04 (0.86–1.26)	1.00 (0.74–1.35)	1.03 (0.76–1.39)	0.86 (0.68–1.09)	1.05 (0.82–1.35)
Pasifika	**0.79 (0.64–0.98)**	0.95 (0.76–1.19)	**1.46 (1.07–2.00)**	**1.55 (1.11–2.16)**	**0.48 (0.36–0.65)**	**0.64 (0.47–0.89)**
Asian	**0.72 (0.60–0.85)**	**0.73 (0.61–0.87)**	1.05 (0.83–1.32)	1.04 (0.82–1.31)	**0.45 (0.35–0.59)**	**0.45 (0.34–0.59)**
MELAA	0.70 (0.38–1.28)	0.76 (0.41–1.40)	1.26 (0.47–3.35)	1.30 (0.48–3.51)	0.49 (0.22–1.10)	0.55 (0.24–1.24)
Full-time worker/non-student (Referent)	1.00	1.00	1.00	1.00	1.00	1.00
Part-time worker/non-student	**0.67 (0.55–0.82)**	**0.69 (0.56–0.84)**	0.98 (0.71–1.34)	0.95 (0.69–1.31)	**0.52 (0.40–0.67)**	**0.52 (0.39–0.67)**
NEET	**0.40 (0.32–0.50)**	**0.43 (0.34–0.54)**	**0.71 (0.51–0.98)**	**0.69 (0.49–0.96)**	**0.26 (0.19–0.35)**	**0.29 (0.21–0.40)**
Full-time student/non-worker	0.92 (0.78–1.07)	0.96 (0.82–1.12)	1.18 (0.95–1.48)	1.19 (0.95–1.49)	**0.71 (0.57–0.89)**	**0.79 (0.63–0.99)**
Full-time student/worker	0.85 (0.72–1.00)	0.88 (0.74–1.04)	0.96 (0.74–1.25)	0.97 (0.74–1.26)	**0.76 (0.60–0.95)**	**0.79 (0.63–0.99)**
Non-disabled (Referent)	1.00	1.00	1.00	1.00	1.00	1.00
Disabled	0.84 (0.67–1.06)	0.92 (0.73–1.16)	0.92 (0.64–1.32)	1.00 (0.69–1.37)	0.81 (0.61–1.09)	0.84 (0.62–1.14)
Low deprivation (Referent)	1.00	1.00	1.00	1.00	1.00	1.00
Medium deprivation	**0.86 (0.75–0.98)**	0.89 (0.78–1.03)	1.10 (0.91–1.34)	1.12 (0.92–1.37)	**0.68 (0.57–0.82)**	**0.71 (0.59–0.86)**
High deprivation	**0.72 (0.62–0.84)**	**0.81 (0.68–0.95)**	1.02 (0.81–1.29)	0.97 (0.76–1.25)	**0.56 (0.45–0.68)**	**0.68 (0.54–0.85)**

Middle Eastern / Latin American / African = MELAA; Not in employment, education, or training = NEET; Unadjusted analyses included socio-demographic characteristics in separate models, whereas the adjusted analyses included socio-demographic characteristics in a single model

^*^Bold text indicate significance levels *p* < 0.05

## Discussion

The findings of this study provide new insight into physical activity levels and differences in a large population of young adults in NZ. Our results demonstrate the need for more effective promotion of physical activity to young adults and the importance of accounting for intersections between socio-demographic characteristics when exploring differences or inequities in physical activity participation. Targeted approaches that consider an array of socio-demographic characteristics are most likely to improve physical activity levels of 18–24 year-olds as opposed to “one-size-fits-all” interventions that account for merely one or two characteristics [13]. The variation in participation differences according to physical activity type (i.e. aerobic vs muscle-strengthening) adds a further layer to how effective physical activity promotion needs to be tailored for different population groups.

The low prevalence of young adults meeting the combined aerobic and muscle-strengthening recommendations in NZ is cause for concern. Although this finding is not surprising given the relatively low physical activity levels among children and youth in NZ [1], intervention during early adulthood has not been prioritised in a physical activity policy setting that currently focuses more on young people [27]. When considering activity types separately, a large proportion of the NZ young adult population are insufficiently active aerobically and even more do not do enough muscle-strengthening activity. The deficit in muscle-strengthening activity levels may reflect a widely acknowledged historical focus on aerobic recommendations in the communication of physical activity guidelines internationally. This provides some impetus for a renewed focus on promoting muscle-strengthening activity [28].

Our results indicate that almost all of those meeting muscle-strengthening recommendations also met the aerobic recommendations. In contrast, approximately 20% of the young adult population in NZ participate in sufficient amounts of aerobic activity, but insufficient amounts of muscle-strengthening activity. Targeting this group of “aerobically-active-only” young adults with effective muscle-strengthening promotion may be a prudent approach to increasing the proportion meeting the combined recommendations, by reaching young adults that are already “primed” to be physically active. However, it does not impact those who are least active and therefore, are likely to gain the most from increasing their participation in any physical activity [29]. It is also a “blanket” approach that does not account for the intersection of the socio-demographic characteristics identified in our results, which could be used to better target and tailor physical activity intervention among young adults in NZ.

Despite widespread assertions that women are less physically active than men [30], our overall results indicated that this was only the case for aerobic activity. This suggests that although it is critical to promote muscle-strengthening activity among women, it does not appear to be more of a priority than for men. However, our findings also highlight that the differences in physical activity participation according to gender is more nuanced than this and promoting different types of physical activity to women may be particularly pertinent in some socio-demographic groups.

Differences in physical activity participation based on the intersection of ethnicity and gender highlight the need to look beyond single demographic characteristics when designing interventions. People of Asian ethnicities were less likely to meet combined physical activity recommendations, but after separating the analyses based on gender it was evident this difference was primarily attributable to deficits in muscle-strengthening activity in Asian women. Furthermore, although it appeared that there were no differences in meeting combined physical activity recommendations among Pasifika peoples, a more nuanced examination of the results indicates that Pasifika women were significantly less active and Pasifika men were significantly more active than their respective reference groups. Without more extensive analyses according to gender and activity type, the high levels of muscle-strengthening activity undertaken by Pasifika men would have masked the clear need for aerobic physical activity intervention with Pasifika women. Importantly, these findings were independent of deprivation status, which in younger age groups has been found to largely explain the physical activity differences observed according to ethnicity [31].

Considerable differences between young men and women were also evident with respect to deprivation status. Among women the likelihood of meeting all physical activity recommendations was lower among those residing in higher deprivation communities, whereas among men, differences were only evident for aerobic activity and this only affected those living in the most deprived areas. Deprivation is a widely recognised determinant of physical activity participation globally that is largely driven by inequities in access to financial resources and local infrastructure [32, 33], but our findings indicate that these inequities are primarily a concern for young women in NZ. This appears to be a continuation of differences in physical activity participation levels according to deprivation and gender that have previously been described in younger age groups in NZ and should be priority when planning intervention [31].

Employment and education status are often linked with socio-economic deprivation and are also known correlates of physical activity participation [34]. However, disentangling the differences based on employment/student status is complex, in part due to overlap in categories and the measures used to define these. Despite these limitations, our results indicate that the overall deficit in both aerobic and muscle-strengthening activity for people who are NEET and part-time employed is primarily driven by the lower participation levels of women in these categories. Similarly, it appears that being enrolled in full-time study is only a negative correlate for physical activity participation for women. However, it is important to note that those who were NEET scored substantially worse than any other employment/student sub-group overall and for both genders. These findings suggest that the greatest need for intervention may not be through traditional workplaces or tertiary institutions, but rather through avenues that reach those not in full-time employment or education. In any case, there should be a greater focus on young women than men when intervening in any of these settings.

Finally, while our analyses suggests that there are no differences based on physical dis/ability, it is worth noting that for this study disability status was based primarily on physical impairments and a relatively small portion of the sample were classified as disabiled [35]. To fully understand differences based on disability among young New Zealanders, efforts must be made to collect data from a more representative sample that also includes those who have intellectual disabilities. This would require changes to the participant recruitment processes and data collection methods used in the Active NZ survey.

Similarly, recruitment of a small number of individuals identifying as gender diverse meant they were excluded from analyses. In addition, other socio-demographic characteristics known to impact physical activity participation among New Zealanders, such as religiosity and sexual orientation, were not assessed [36, 37]. In light of the differences we identified for the socio-demographics included in the analyses, it is worth considering what additional characteristics could be assessed and included in future research. For example, targeted recruitment of more gender diverse individuals would allow for inclusion in analyses [38].

Despite these limitations, the size of this dataset is a strength of the current study. The inclusion of multiple physical activity measures that capture different components of the physical activity recommendations is also noteworthy and not common place in international surveillance systems [39]. However, future iterations of the survey should include measures that capture information about physical activity beyond the leisure domain. For example, understanding physical activity across the transport, domestic and occupational domains would help build a clearer picture of exactly where differences exist among young adults and across the lifespan. Supplementing the use of self-report physical activity measurement tools that are widely known to overestimate participation rates with objective measures of movement would also align surveillance of physical activity in NZ with best practice internationally [40]. Finally, incorporating variables pertaining local policies, physical environments and socio-cultural factors that directly or indirectly impact physical activity would also help identify the determinants of inequities beyond socio-demographic characteristics [41].

In summary, given physical activity typically declines over the course of the lifespan and young adults tend to over-report their physical activity participation [42], our findings are of public health concern. Specifically, participating in insufficient physical activity has implications for cardiovascular disease, cancer, and mental illnesses that are the leading causes of morbidity and mortality globally [26]. Less than a half of young adults in this study met both aerobic and muscle-strengthening recommendations, and considerable differences demonstrate the need for urgent action to promote physical activity in this age group in NZ. This is particularly the case for young adults not currently employed or enrolled in tertiary education and young women who live in deprived areas, or identify as Asian or Pasifika. Finally, it is evident that physical activity surveillance systems in NZ need to be more inclusive of several minority groups (e.g. disabled people) and should supplement existing self-report measures with more up-to-date methods of assessing movement (e.g. accelerometry).

## Data Availability

The data that support the findings of this study are available from Sport New Zealand Ihi Aotearoa but restrictions apply to the availability of these data, which were used under license for the current study, and so are not publicly available. Data are however available from the authors upon reasonable request and with permission of Sport New Zealand Ihi Aotearoa. Further information on the dataset is available here—https://sportnz.org.nz/research-and-insights/surveys-and-data/active-nz/.
